# Development of Novel Tetracycline and Ciprofloxacin Loaded Silver Doped Hydroxyapatite Suspensions for Biomedical Applications

**DOI:** 10.3390/antibiotics12010074

**Published:** 2022-12-31

**Authors:** Daniela Predoi, Simona-Liliana Iconaru, Mihai-Valentin Predoi, Nicolas Buton

**Affiliations:** 1 National Institute of Materials Physics, 405A Atomistilor Street, 077125 Magurele, Romania; 2 Department of Mechanics, University Politehnica of Bucharest, BN 002, 313 Splaiul Independentei, Sector 6, 060042 Bucharest, Romania; 3 HORIBA Jobin Yvon S.A.S., 6-18, Rue du Canal, CEDEX, 91165 Longjumeau, France

**Keywords:** silver, hydroxyapatite, antibiotics, suspensions, antimicrobial activity

## Abstract

The objective of this study consisted of the development of new materials with antimicrobial properties at the nanometric scale that could lead to an increase in therapeutic efficacy and reduction of toxic side effects. This work focuses on obtaining and characterizing stable suspensions with narrow size distribution with antimicrobial properties. The stability of the suspensions obtained by an adapted co-precipitation method was evaluated by ultrasonic measurements. The size and size distribution of the particle populations were determined using scanning electron microscopy (SEM), and dynamic light scattering (DLS). Both methods of analysis showed a narrow distribution of particles. DLS gave a monomodal distribution with hydrodynamic diameters around 38 nm for ciprofloxacin embedded in silver doped hydroxyapatite (AgHA-C) and 45.7 nm for tetracycline embedded in silver doped hydroxyapatite (AgHA-T). The average diameters calculated from SEM were 17 nm for AgHA-C and 19 nm for AgHA-T. Both Ciprofloxacin and Tetracycline influenced the hydroxyapatite structure, which led to the appearance of new vibrational bands characteristic of the specific chemical composition in the FTIR spectrum. The antimicrobial properties of the AgHA-C and AgHA-T suspensions were assessed using the most common reference microbial strains *Staphylococcus aureus* ATCC 25923, *Escherichia coli* ATCC 25922, and *Candida albicans* ATCC 10231. The results of the in vitro antimicrobial assays determined that the AgHA-C and AgHA-T suspensions exhibited exceptional antimicrobial activity. Moreover, the data revealed that the antimicrobial activity increased with the increase of the incubation time.

## 1. Introduction

In the last years, the increase of pathogens’ resistance against conventional antibiotics has been the principal cause of the apparition of dangerous health problems worldwide. Microbial resistance was deemed one of the most pressing issues against the majority of antibiotics around the globe [1]. Over the years, numerous attempts were made towards the development of safe and more effective protocols in order to overcome the problems risen due to the apparition of drug resistant microorganisms. The resistance to conventional antibiotics involves mechanisms like reduced uptake or increased efflux of antibiotics out of the microbial cell. These phenomena have the ability to reduce the antibiotic contents from the bacterial cell leading to the inhibition of the bacterial cell toxicity and the enzymatic modification, thus to the inactivation of the antibiotic [2]. As a response to this worldwide crisis, researchers have turned their attention to the development of novel materials which possess antimicrobial activity on their own or which could be used as enhancers for the antimicrobial properties of conventional antibiotics. During the years, hydroxyapatite (HA) was one of the most studied materials for biomedical applications due to its proven biocompatibility and its unique biological properties [3,4,5,6,7,8]. Nowadays, HA is known as a promising material and is widely employed in medicine, dentistry, drug delivery, and implantology [9,10] due to its similarity with the main inorganic component found in mineralized tissues [11]. Research involving HA is currently trying to both improve its biocompatibility and provide HA supplementary properties [12]. One of the preferred modes of improving the properties of HA is the incorporation of different ions into the structure of HA. This approach contributes substantially to the improvement of HA properties such as crystal size, agglomeration tendency, solubility, and biological properties like osteoconduction, osteointegration, biocompatibility as well as antimicrobial activity [13,14,15,16]. The ubiquitous structure of HA allows a large number of ionic substitutions of calcium ions from its structure with ions such as zinc, selenium, strontium, magnesium, europium, cerium, samarium, etc [13,14,15,16,17,18]. Previous studies regarding the use of silver as a dopant for HA reported [17,18,19,20] demonstrated that the incorporation of silver ions into HA lattice confers excellent antimicrobial activity to HA materials while also having good biocompatible properties. Moreover, recently, researchers proposed the enhancement of HA properties by employing physical or chemical binding of drugs [20,21,22,23]. Studies describe using HA as a delivery system for various drugs, such as antiresorptive drugs, anticancer medication, and antibiotics [20,21,22,23]. The procedure of locally releasing antibiotics, with the help of bone cement impregnation or PMMA chains, has been introduced in early 1970 in orthopedic surgery and has been deemed to help prevent the apparition of post-operatory infections [24]. Since then, improved solutions for the development of novel materials were extensively studied. Studies have shown that a disadvantage of using calcium phosphates as drug delivery systems for the treatment of bone infections is the rapid release of antibiotics. Over the years various methods for improving these aspects have been developed [25]. This process occurs because, in most cases, the drug loading mechanism is adsorption from solutions. This mechanism leads to rapid drug release over several days. In their paper, Stigter et al. [26] reported a comparison between the efficiency of the incorporation of different antibiotics into carbonated HA coatings. Their study revealed that the incorporation rate of the antibiotic was dependent on the chemical structure of the antibiotic. Another study regarding the loading of HA powders with different porosity with vancomycin, ciprofloxacin, and gentamicin was reported by Chai et al. [27]. The results of their study concluded that the adsorption of antibiotics was significantly influenced by both the HA porosity and the antibiotic. Moreover, Chai et al. [27] demonstrated in their study that materials demonstrated strong antibacterial activity against different bacterial strains such as *S. aureus*, Staphylococcus epidermidis, and *E. coli*. In our study, we have decided to load silver doped hydroxyapatite suspensions with one of the most known antibiotics, ciprofloxacin, and tetracycline. Ciprofloxacin belongs to the fluoroquinolones class of antibiotics and is widely used for treating bacterial infections, being also the first choice in the treatment of bacterial keratitis. It has been reported to be effective in the treatment of corneal ulcers caused by methicillin resistant *S. aureus* and also by different strains of *S. aureus* that are resistant to other antibiotics, such as vancomycin and cefazolin [28,29,30]. Tetracycline is a well-known broad-spectrum antibiotic, which was reported to be effective against gram-positive cocci and some gram-negative organisms and also against rickettsia and chlamydia [31]. At present, tetracycline is employed in the treatment of infections determined by bacteria like pneumonia, respiratory tract infections, skin infections, eye and lymphatic, infections as well as infections found in the intestinal, genital, and urinary systems [31,32]. Silver is well known for its antimicrobial properties and is one of the most used and studied agents due to its broad spectrum of action and low toxicity at low concentrations [19,33,34], therefore, the use of metallic ions such as silver as dopant confer to hydroxyapatite the ability to fight against gram-positive (*Staphylococcus aureus*, *Streptococcus mutans*, *Bacillus cereus*) [20,35], gram-negative (*Escherichia coli*, *Aggregatibacter actinomycetemcomitans*, *Fusobacterium nucleatum*) [36] bacterial strains and fungi (*Candida albicans*) [37]. Moreover, the loading of antibiotics such as tetracycline and ciprofloxacin [38] onto silver doped hydroxyapatite would allow the obtaining of novel material with higher antimicrobial activity and excellent biocompatible properties. In this context, the aim of this study is the development of stable novel silver doped hydroxyapatite suspensions loaded with well-known antibiotics like tetracycline and ciprofloxacin for biomedical applications. The achievement of this goal will allow the development of new stable suspensions possessing both excellent antimicrobial and biocompatible properties. The stability of the AgHA-C and AgHA-T suspensions obtained by an adapted co-precipitation method was investigated by ultrasonic measurements. Moreover, SEM and DLS techniques were used to determine the morphology and size distribution of the particle populations. FTIR studies were also used to determine the influence of ciprofloxacin and tetracycline on the HA structure. On the other hand, the antimicrobial properties of the AgHA-C and AgHA-T suspensions were assessed against *Staphylococcus aureus* ATCC 25923, *Escherichia coli* ATCC 25922, and *Candida albicans* ATCC 10231. The results showed that the development of HA composites loaded with antibiotics could have the ability to broaden the medical application of hydroxyapatite and allow targeted drug delivery to pathological zones of interest and will also be able to provide a long-term therapeutic action.

## 2. Results and Discussion

**Figure 1:**
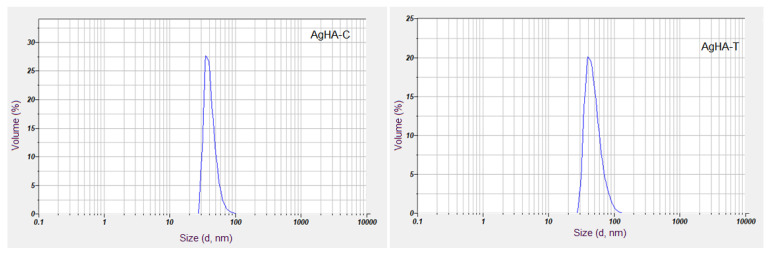
The volume size distribution analyzed with DLS for AgHA-C (**left**) and AgHA-T (**right**) suspensions.

According to existing studies [39], DLS is one of the most valuable techniques for evaluating the hydrodynamic diameter and the distribution of particles in the solution. Thus, the DLS technique uses a laser beam to illuminate a suspension of particles or molecules undergoing Brownian motion [40]. In this study, DLS was used to determine the hydrodynamic diameter of the particles in aqueous suspension as well as their dispersion. The volume-based distribution, as revealed in Figure 1, clearly shows that the AgHA-C and AgHA-T suspensions do not present aggregates.

The volume size distribution shows that the analyzed samples consisted of small particles of approximately 38 nm (AgHA-C) and 45.7 nm (AgHA-T). The mean weighted hydrodynamic diameter (Z-Average), depending on the intensity of the two analyzed samples was 44.38 nm (AgHA-C) and 56 nm (AgHA-T). The polydispersity index (P.I.) values were 0.186, for AgHA-C and 0.201 for AgHA-T.

**Figure 2:**
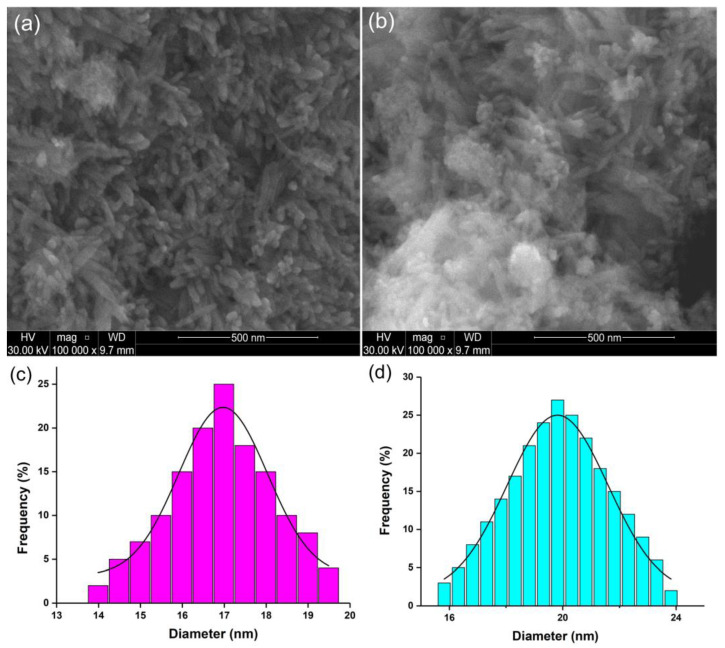
SEM micrograph of AgHA-C (**a**) and AgHA-T (**b**) suspensions. Mean diameters calculated from SEM analysis for AgHA-C (**c**) and AgHA-T (**d**) suspensions.

The SEM micrographs show the morphology and particle size of the synthesized AgHA-C and AgHA-T suspensions (Figure 2a,b). AgHA-C particles in suspension have an ellipsoidal shape. On the other hand, AgHA-T particles in suspension have an ellipsoidal shape slightly different from that of AgHA-C, having a slight tendency to agglomerate. The mean diameters calculated from SEM analysis (D_SEM_) by counting about 500 particles was 17 ± 2 nm for AgHA-C suspension (Figure 2c). For AgHA-T suspension, D_SEM_ was 19 ± 2 nm (Figure 2d). In the case of both analyzed samples, the size distribution is narrow. Despite the fact that the visualization of particles with scanning electron microscopy is widely applied because it provides information on the morphology of individual particles, it has the disadvantage of being able to see only the core of the studied particles. On the other hand, DLS measures time-dependent fluctuations in the intensity of light scattered from a suspension of particles in random Brownian motion providing information on the hydrodynamic size. The hydrodynamic size is the size of the nanoparticle plus the liquid layer around the particle. The calculated ratio of the hydrodynamic diameter obtained from DLS analysis and diameter obtained by SEM was D_H_/D_SEM_ was 2.24 for AgHA-C suspension while the D_H_/D_SEM_ for AgHA-T suspension was 2.34. 

According to previously developed studies [41], the difference between the sizes of suspended particles is given by the fact that following DLS measurements we have distributions of diffusion coefficients that are transformed into distributions of hydrodynamic diameters (D_H_). Moreover, in agreement with previous studies [42], in the case of heterogeneous populations, the weighting procedure is different, which leads to inevitable differences between the two analysis methods (DLS and SEM). Moreover, the elemental composition estimated from X-ray Energy Dispersive Spectroscopy (EDS) analysis was presented in Table 1. The EDS results confirm the presence of both silver ions in the samples, as well as the constituent elements of the hydroxyapatite and the two antibiotics, ciprofloxacin and tetracycline, respectively.

**Figure 3:**
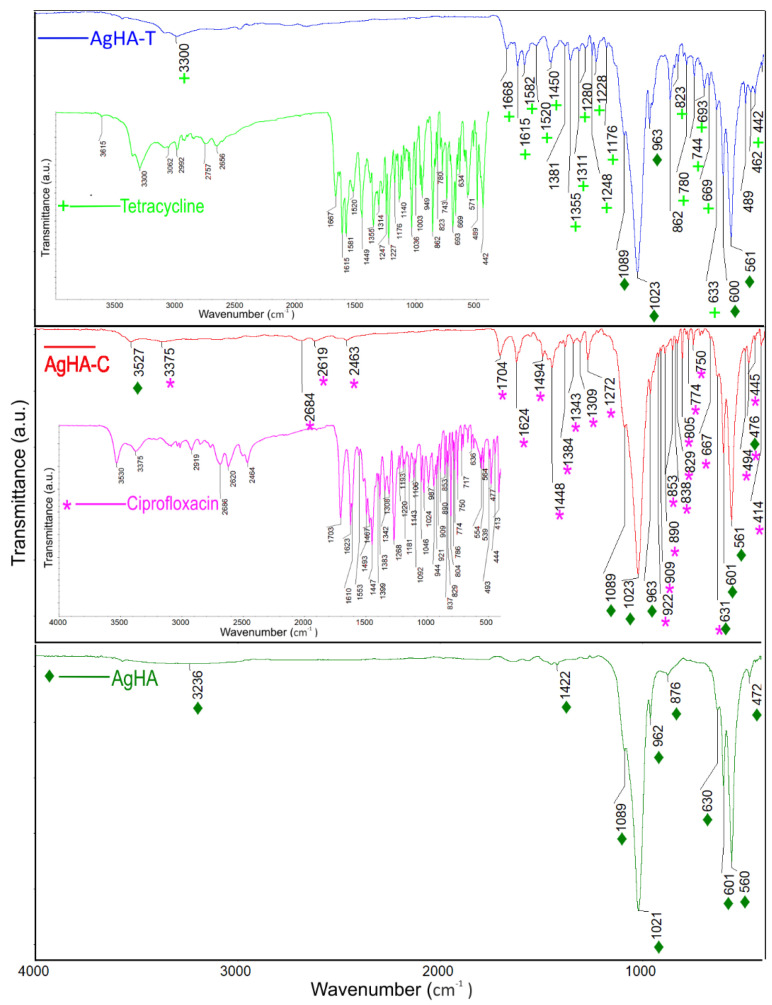
FTIR spectra of AgHA, AgHA-C and AgHA-T as well as the spectra of the two antibiotics, Ciprofloxacin and Tetracycline. ♦—peaks associated to HA; ∗—peaks associated to Ciprofloxacin; +—peaks associated to Tetracycline.

The infrared transmission spectra of AgHA, AgHA-C, and AgHA-T were performed to confirm the presence of antibiotics (Figure 3). The spectra of ciprofloxacin and tetracycline were acquired to see the peaks that correspond to them (Figure 3). The vibration bands that are characteristic of the pure hydroxyapatite structure are found in all three samples (AgHA, AgHA-C, and AgHA-T) analyzed. The bands determined at 472 cm^−1^, 560 cm^−1^, 601 cm^−1^, and 1022 cm^−1^ are associated with the PO_4_^3−^ phosphate group and highlight the bending modes of the O-P-O bonds [43,44,45,46]. The vibration bands determined at 962 cm^−1^ and 1089 cm^−1^ associated with the PO_4_^3−^ phosphate group are characteristic of the non-degenerate symmetric stretching mode of the P-O bond and the triple degenerate symmetric stretching mode of the P-O bond [43,44,45,46,47], respectively. The vibration bands that are characteristic of the pure hydroxyapatite structure are found in all three samples (AgHA, AgHA-C, and AgHA-T) analyzed. The bands determined at 472 cm^−1^, 560 cm^−1^, 601 cm^−1^, and 1022 cm^−1^ are associated with the PO_4_^3−^ phosphate group and highlight the bending modes of the O-P-O bonds [43,44,45,46]. The vibration bands determined at 962 cm^−1^ and 1089 cm^−1^ associated with the PO_4_^3−^ phosphate group are characteristic of the non-degenerate symmetric stretching mode of the P-O bond and the triple degenerate symmetric stretching mode of the P-O bond [43,44,45,46,47], respectively. The vibrational band at 631 cm^−1^ corresponds to the presence of the hydroxyl group in the AgHA, AgHA-C, and AgHA-T analyzed samples [43]. 

**Figure 4:**
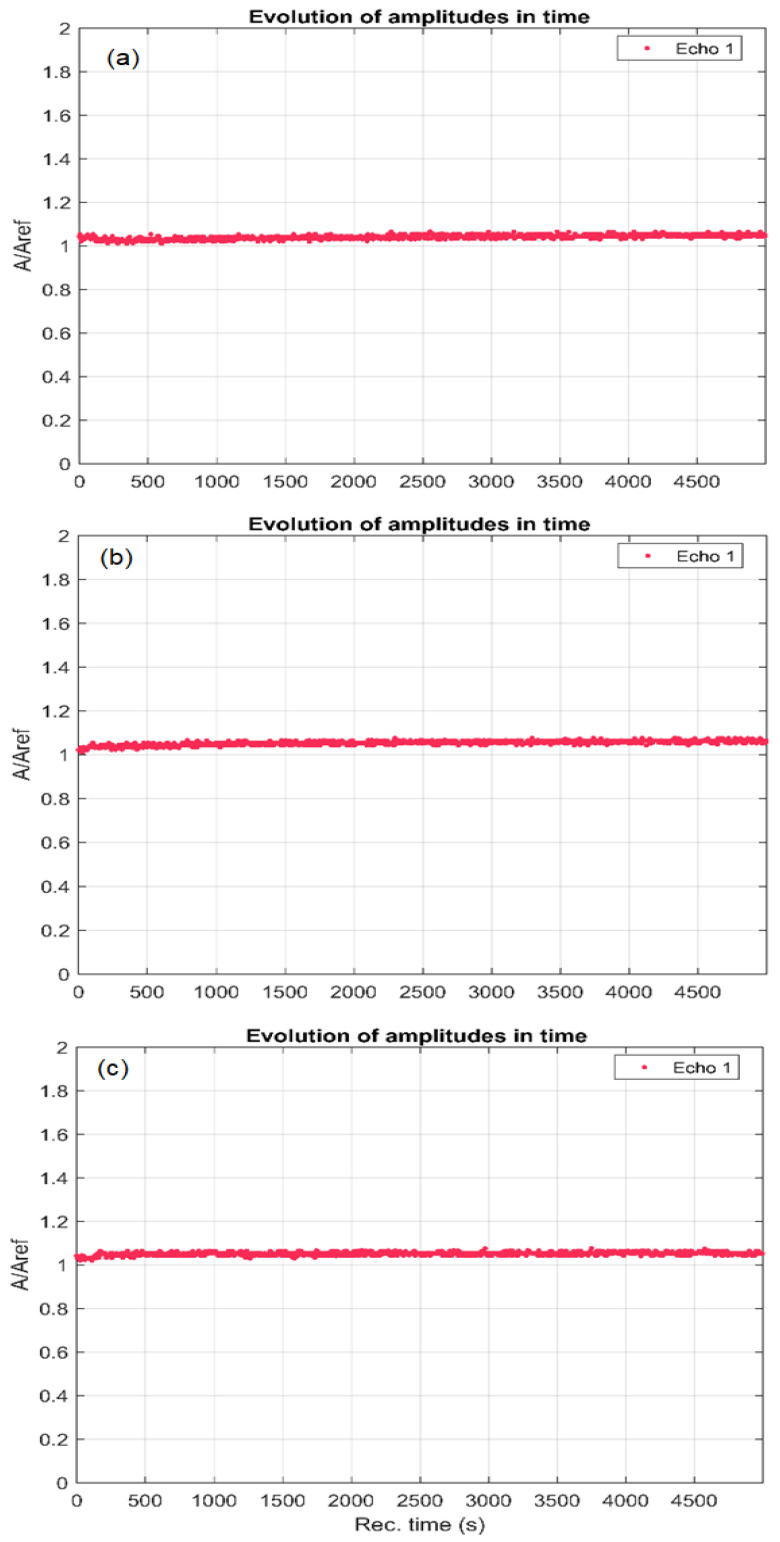
Evolution in time of the signal relative amplitude of samples AgHA (**a**), AgHA-C (**b**) and AgHA-T (**c**).

The characteristic bands of carbonates at 876 cm^−1^ and 1422 cm^−1^ [48] were also detected in the FT-IR spectrum. The vibrational bands identified at 3236 and 1623 cm^−1^ were assigned to the O-H stretching and bending vibrations of water, respectively [49]. Additional bands observed in the characteristic spectrum of AgHA-C are associated with functional groups characteristic of Ciprofloxacin. The characteristic spectrum of Ciprofloxacin was inserted, and the characteristic peaks have been marked with a magenta star. In FTIR spectra the characteristic peaks of ciprofloxacin were found between 3530 and 3450 cm^−1^, which was assigned to stretching vibration of OH groups and intermolecular hydrogen bonding. Other bands at 2919–2686 cm^−1^ represented C-H stretching, mainly υ = C-H [50,51]. The N-H stretching frequencies characteristic of ciprofloxacin was observed at 2464 cm^−1^ [50,51,52,53]. The band at 1750 to 1703 cm^−1^ represented the carbonyl C=O stretching i.e., υC=O. Moreover, the bands at 1623 cm^−1^ and 1467 cm^−1^ represented both asymmetric and symmetric stretching vibration of the O-C-O group, respectively. The band from 1553 to 1340 cm^−1^ represented υC-O and at 1308 to 1263 cm^−1^ suggested bending vibration of the O-H group [50,51,52,53]. The peaks at 1450 and 1400 cm^−1^ were for υC-O/δO-H. The band at 1268 to 1181 cm^−1^ was due to υC-O-C of acrylates [50,53]. An absorption peak between 1106 and 1046 cm^−1^ was assigned to the C-F group [50,51,52,53]. In addition, the band at 1024 to 987 cm^−1^ was assigned to υC-F [50,51,52,53]. The band between 853 to 804 cm^−1^ was for out-of-plane bending of =C-H i.e., δ = C-H [50,53]. On the other hand, the additional bands observed in the characteristic spectrum of AgHA-T are associated with functional groups characteristic of Tetracycline. The characteristic Tetracycline spectrum was inserted, and the characteristic peaks have been marked with a green cross. In the FTIR spectra of tetracycline, there are bands corresponding to the aromatic ring stretching vibrations (1449 cm^−1^, 1581 cm^−1^, 1615 cm^−1^, 1667 cm^−1^) [54]. The band found at around 1355 cm^−1^ was associated with either C-O stretching, symmetric CH_3_ bending mode, or the terminal dimethyl bending vibrational mode [54], while the bands from around 1227 and 1247 cm^−1^ were assigned to the C-N stretching mode, and the C-C stretching mode [54]. The peaks of tetracycline were also highlighted by the presence of bands b [55] between 669 cm^−1^ and 949 cm^−1^ which are associated with the aromatic =C-H deformations [55]. On the other hand, the bands from 489 cm^−1^, 669 cm^−1^, and 693 cm^−1^ correspond to the out-of-plane aromatic ring deformation, while the band from 634 cm^−1^ evidences the in-plane ring deformation [55]. As can be seen in Figure 3, the structures of the two antibiotics are complex, being difficult to distinguish the vibrational bands associated with each functional group, since in the same spectral region bands characteristic of the vibrations of different chemical bonds can be identified. On the other hand, it can be observed that the structure of hydroxyapatite doped with silver was influenced by the presence of the antibiotic, which led to the creation of new vibrational bands that are characteristic of the specific chemical composition. The additional bands observed due to the presence of antibiotics are much more attenuated than those observed in the reference spectra of the two antibiotics. The presence of additional bands associated with the antibiotics suggests a good interaction between them and the apatite structure. To evaluate the stability of AgHA-C and AgHAp-T suspensions, ultrasonic measurements were made. The advantage of this technique is that the stability of the analyzed suspensions can be evaluated without diluting them. Three suspensions were analyzed by ultrasonic waves: AgHA (sample A), AgHA-C (sample B, containing Ciprofloxacin antibiotic), and AgHA-T (sample C with Tetracycline antibiotic). A volume of 100 mL of each sample has been steered for 15 min at 500 rot/min in a special cubic vessel. The vessel has coaxially opposed ultrasonic transducers of central frequency 25 MHz. Immediately after stopping the steering, the ultrasonic signals transmitted from one transducer to the other, are recorded every 5 s, for a total duration of 5000 s. The amplitudes of the transmitted signals are determined as ratios to the amplitudes in double distilled water, which is taken as reference liquid. Figure 4 shows the relative amplitudes of the three samples.

The characteristic bands of carbonates at 876 cm^−1^ and 1422 cm^−1^ [48] were also detected in the FT-IR spectrum. The vibrational bands identified at 3236 and 1623 cm^−1^ were assigned to the O-H stretching and bending vibrations of water, respectively [49]. Additional bands observed in the characteristic spectrum of AgHA-C are associated with functional groups characteristic of Ciprofloxacin. The characteristic spectrum of Ciprofloxacin was inserted, and the characteristic peaks have been marked with a magenta star. In FTIR spectra the characteristic peaks of ciprofloxacin were found between 3530 and 3450 cm^−1^, which was assigned to stretching vibration of OH groups and intermolecular hydrogen bonding. Other bands at 2919–2686 cm^−1^ represented C-H stretching, mainly υ = C-H [50,51]. The N-H stretching frequencies characteristic of ciprofloxacin was observed at 2464 cm^−1^ [50,51,52,53]. The band at 1750 to 1703 cm^−1^ represented the carbonyl C=O stretching i.e., υC=O. Moreover, the bands at 1623 cm^−1^ and 1467 cm^−1^ represented both asymmetric and symmetric stretching vibration of the O-C-O group, respectively. The band from 1553 to 1340 cm^−1^ represented υC-O and at 1308 to 1263 cm^−1^ suggested bending vibration of the O-H group [50,51,52,53]. The peaks at 1450 and 1400 cm^−1^ were for υC-O/δO-H. The band at 1268 to 1181 cm^−1^ was due to υC-O-C of acrylates [50,53]. An absorption peak between 1106 and 1046 cm^−1^ was assigned to the C-F group [50,51,52,53]. In addition, the band at 1024 to 987 cm^−1^ was assigned to υC-F [50,51,52,53]. The band between 853 to 804 cm^−1^ was for out-of-plane bending of =C-H i.e., δ = C-H [50,53]. On the other hand, the additional bands observed in the characteristic spectrum of AgHA-T are associated with functional groups characteristic of Tetracycline. The characteristic Tetracycline spectrum was inserted, and the characteristic peaks have been marked with a green cross. In the FTIR spectra of tetracycline, there are bands corresponding to the aromatic ring stretching vibrations (1449 cm^−1^, 1581 cm^−1^, 1615 cm^−1^, 1667 cm^−1^) [54]. The band found at around 1355 cm^−1^ was associated with either C-O stretching, symmetric CH_3_ bending mode, or the terminal dimethyl bending vibrational mode [54], while the bands from around 1227 and 1247 cm^−1^ were assigned to the C-N stretching mode, and the C-C stretching mode [54]. The peaks of tetracycline were also highlighted by the presence of bands b [55] between 669 cm^−1^ and 949 cm^−1^ which are associated with the aromatic =C-H deformations [55]. On the other hand, the bands from 489 cm^−1^, 669 cm^−1^, and 693 cm^−1^ correspond to the out-of-plane aromatic ring deformation, while the band from 634 cm^−1^ evidences the in-plane ring deformation [55]. As can be seen in Figure 3, the structures of the two antibiotics are complex, being difficult to distinguish the vibrational bands associated with each functional group, since in the same spectral region bands characteristic of the vibrations of different chemical bonds can be identified. On the other hand, it can be observed that the structure of hydroxyapatite doped with silver was influenced by the presence of the antibiotic, which led to the creation of new vibrational bands that are characteristic of the specific chemical composition. The additional bands observed due to the presence of antibiotics are much more attenuated than those observed in the reference spectra of the two antibiotics. The presence of additional bands associated with the antibiotics suggests a good interaction between them and the apatite structure. To evaluate the stability of AgHA-C and AgHAp-T suspensions, ultrasonic measurements were made. The advantage of this technique is that the stability of the analyzed suspensions can be evaluated without diluting them. Three suspensions were analyzed by ultrasonic waves: AgHA (sample A), AgHA-C (sample B, containing Ciprofloxacin antibiotic), and AgHA-T (sample C with Tetracycline antibiotic). A volume of 100 mL of each sample has been steered for 15 min at 500 rot/min in a special cubic vessel. The vessel has coaxially opposed ultrasonic transducers of central frequency 25 MHz. Immediately after stopping the steering, the ultrasonic signals transmitted from one transducer to the other, are recorded every 5 s, for a total duration of 5000 s. The amplitudes of the transmitted signals are determined as ratios to the amplitudes in double distilled water, which is taken as reference liquid. Figure 4 shows the relative amplitudes of the three samples.

**Figure 5:**
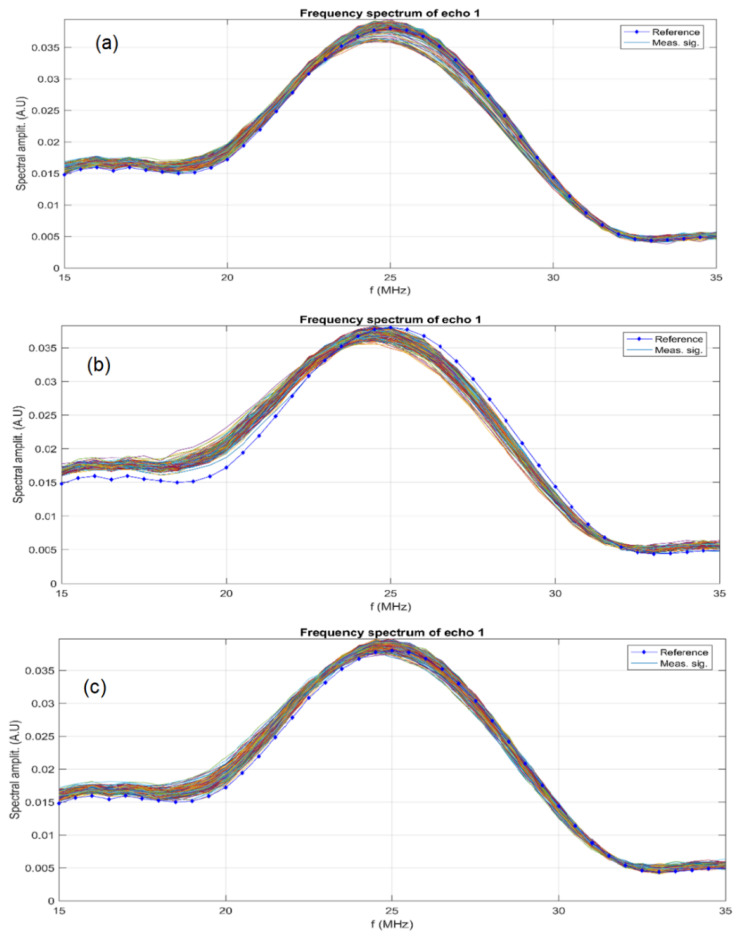
Frequency spectra of all recorded signals and of the reference liquid AgHA (**a**), AgHA-C (**b**) and AgHA-T (**c**) samples from top to bottom.

Sample AgHA (Figure 4a) shows a small initial (time < 100 s) reduction of the relative amplitude, followed by a steady slow increase of amplitude. Samples AgHA-C (Figure 4b) and AgHA-T (Figure 4c) have a small initial rise in amplitudes followed by steady slow increasing amplitude AgHA-C (Figure 4b) and a constant amplitude AgHA-T (Figure 4c). The ultrasonic signals transmitted through the three samples are transformed in frequency spectra, and superposed for all 1000 recorded signals (Figure 5). The maximum amplitudes are near the central frequency of 25 MHz, as expected, and proved by the curves obtained for the reference liquid.

**Figure 6:**
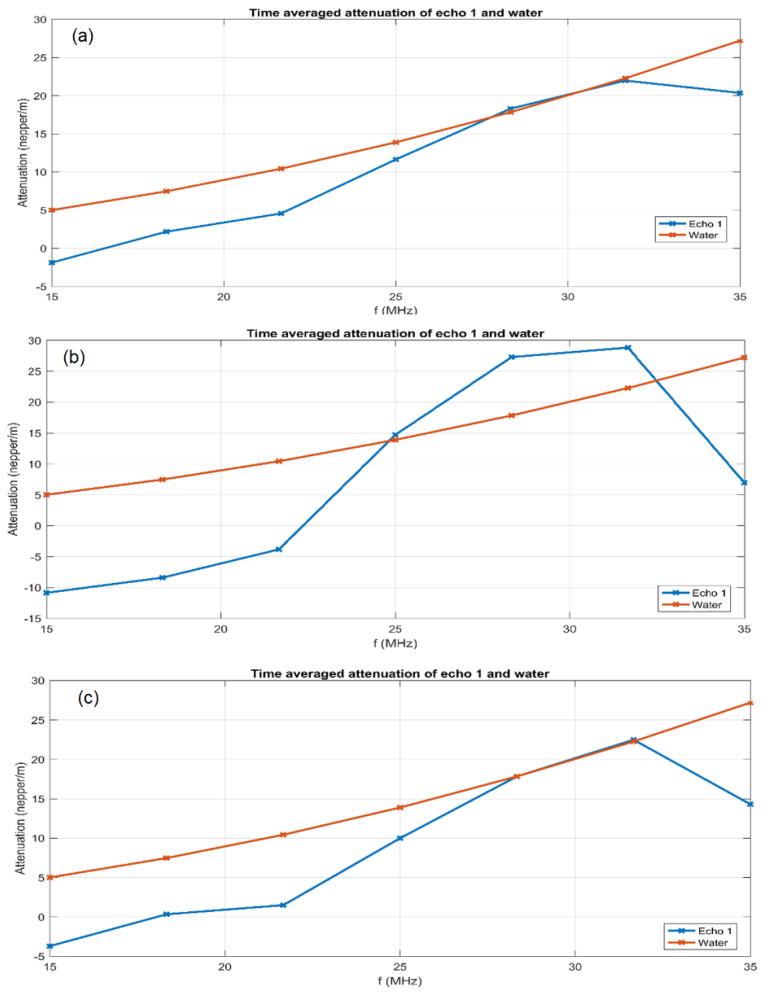
Attenuation of ultrasonic signals vs. frequency for the three samples AgHA (**a**), AgHA-C (**b**), and AgHA-T (**c**).

However, there are differences between samples AgHA (Figure 5a) which show more spread curves around 25 MHz, indicating a more rapid change in sample properties, by faster sedimentation. This remark is not confirmed for sample AgHA-T (Figure 5c), which indicates higher stability for this last sample. Sample AgHA-C (Figure 5b) has higher amplitudes than in reference fluid for frequencies below the central frequency and lower amplitudes above this central frequency (25 MHz). This remark indicates a weak attenuation at frequencies below the central frequency, which can only be explained by the solid bond between the metallic component and HAp, provided by the added ciprofloxacin. On the other hand, at frequencies above the central frequency, the signals are weaker than in water, indicating a stronger dissipation of energy, most likely due to the HAp. These remarks are confirmed by the attenuation of signals at a range of frequencies centered on 25 MHz (Figure 6).

All three samples indicate lower attenuation than in the reference liquid, in the lower frequency range but also the highest frequencies of the computed spectrum. This can be explained by the presence of Ag ions. Samples AgHA (Figure 6a) and AgHA-T (Figure 6c) only reach the attenuation in water at around 30 MHz. Only sample AgHA-C (Figure 6b) is influenced by the ciprofloxacin, exhibiting a higher attenuation than in the reference liquid for the 25–32 MHz frequency range, due to possible resonating behavior in this frequency range. The stability parameter $S=\bar{\frac{dA}{Adt}}$, averaged for sample AgHA (Figure 6a): S_A_ = 9.98467e−07 (1/s), for AgHA-C (Figure 6b): S_B_ = 1.93849e−05 (1/s), and for AgHA-T (Figure 6c): S_C_ = 1.00893e−05 (1/s). All values indicate good stability, but the stability of sample AgHA (Figure 6a) is outstanding, being very close to S_w_ = 0 for the reference liquid.

The stability of the AgHA-C and AgHAp-T suspensions was estimated following ultrasound measurements performed both for the standard solution (double-distilled water considered as the reference fluid) and for the suspensions of AgHA-C and AgHAp-T in the resulting form (without achieving their dilution). The results regarding the stability of AgHA-C and AgHAp-T suspensions were evaluated by comparison with double-distilled water (known as the most stable suspension). Thus, the values obtained from ultrasonic measurements for the stability parameter of AgHA-C and AgHAp-T suspensions clearly highlighted their stability in relation to double-distilled water.

**Figure 7:**
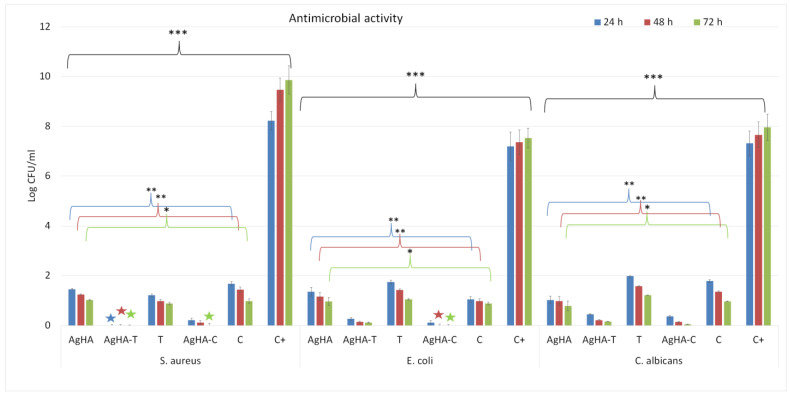
Graphical representation of the logarithmic values of colony forming units (CFU)/mL of *S. aureus* ATCC 25923, *E. coli* ATCC 25922, and *C. albicans* ATCC 10231 microbial strains after 24, 48 and 72 h of incubation with AgHA, AgHA-T and AgHA-C suspensions. The results are depicted as mean ± standard deviation from 3 independent experiments. The statistical analysis was performed using one-way ANOVA. The *p*-values indicated correspondence are * *p* ≤ 0.003, ** *p* ≤ 0.001, *** *p* ≤ 0.0001. The stars depict the bactericidal activity of the samples.

The antimicrobial activity of AgHA, AgHA-C, and AgHA-T suspensions as well as tetracycline (T) and ciprofloxacin (C) was assessed using *S. aureus* ATCC 25923, *E. coli* ATCC 25922, and *C. albicans* ATCC 10231 microbial strains. The antimicrobial assays were performed for three different incubation time intervals (24, 48, and 72 h). The results obtained from three independent antimicrobial experiments were presented as mean ± standard deviation and are depicted in Figure 7. The statistical analysis was performed using one-way ANOVA. The results of the antimicrobial studies revealed that AgHA, AgHA-C, and AgHA-T suspensions exhibited strong inhibitory effects against the tested microbial strain compared both to the control (C+) microbial suspensions as well as tetracycline and ciprofloxacin samples. Moreover, the data suggested that the antimicrobial activity of the AgHA, AgHA-C, and AgHA-T suspensions was influenced by the incubation time and also by the antibiotic used as well as the microbial strain. In addition, the results of the antimicrobial assays depicted that the two investigated antibiotics presented only bacteriostatic effects, while the AgHA-C and AgHA-T samples also exhibited bactericidal properties in the case of *S. aureus* and *E. coli* bacterial strains. Moreover, the results depicted that the antimicrobial activity of AgHA was enhanced by the presence of the two antibiotics for all the tested microbial strains. These results evidenced that even though the silver ions confer antimicrobial activity to the HA composite, the antimicrobial effects could be enhanced by also loading them with an antibiotic. 

The antimicrobial studies performed on the AgHA, AgHA-C, and AgHA-T suspensions highlighted that the loading of AgHA with tetracycline determined a complete bactericidal effect of the suspensions against *S*. *aureus* and an increase of the bacteriostatic activity in the case of *E. coli* and also an increase in the antifungal inhibitory effect in the case of *C. albicans* (the bactericidal effects are marked on the graphic with stars). Those results are in good agreement with previously reported studies regarding the antimicrobial effects of tetracycline [56,57,58]. In addition, the results of the antimicrobial experiments regarding the AgHA loaded with ciprofloxacin also depicted that the ciprofloxacin determined a bactericidal effect of AgHA against *S. aureus* after 72 h of incubation and against *E. coli* after 48 h of incubations. More than that, the results also showed that the ciprofloxacin added to the AgHA also determined an increase in the antifungal activity which was greater than in the case of AgHA-T suspensions. These results are also in agreement with previously reported studies regarding the ciprofloxacin effects on different microbial strains [59,60,61,62]. In their studies, Ibraheem et al. [38] reported that loading chemically synthesized AgNPs with ciprofloxacin resulted in a material with enhanced antibacterial properties against pathogenic bacterial isolates, i.e., *S. aureus*, *A. baumannii*, and *S. marcescens*, compared to both bare silver nanoparticles as well as molecularly free ciprofloxacin. Information about reported studies regarding the development of novel materials with tetracycline and ciprofloxacin are depicted in Table 2. 

Even though there is still scarce information regarding the mechanism involved in the antimicrobial properties of nanomaterials, over the years great efforts have been made to gather results that could lead to a complex understanding of this phenomenon. In this light, there are various reported mechanisms that could possibly explain the antimicrobial activity of the individual compounds that were used for the development of the AgHA, AgHA-C, and AgHA-T suspensions such as reactive oxygen species (ROS) production, disruption of the cell wall and cytoplasmic membrane, perforation of the membrane, respiratory enzymes deactivation, denaturation of ribosomes, interrupting adenosine triphosphate production, deoxyribonucleic acid (DNA) modification in the case of silver ions. On the other hand, the mechanism that confers antibacterial properties to ciprofloxacin, is correlated with the interfering with the replication and transcription of DNA by inhibiting the bacterial DNA gyrase/topoisomerase II and DNA topoisomerase IV, while tetracycline’s mechanism of action is due to the fact that this compound has the ability to bind reversibly to the 30S ribosomal subunit to a position that leads to the block of the binding of the aminoacyl-tRNA to the acceptor site on the mRNA-ribosome complex [61,62,63,64]. Nonetheless, there is still new information to be gained regarding the mechanism of actions of these compounds that could appear due to the synergistic effects that occur in the composite samples. The strong antimicrobial activities of these types of materials are attributed to both the silver ions as well as the antibiotics. Various studies reported that these types of materials exhibit strong antimicrobial properties due to different processes, and in order to lower their toxicity they are usually used in low concentrations [63,64]. One of the possible mechanisms involved in the case of silver ions and antibiotics is the chelation and interaction of silver with the antibiotic, which results in structures formed of an Ag metal nano-core, surrounded by antibiotic molecules, which has the ability to promote the continuous persistence and release of the antibiotic molecules at predestined sites of the microbial infections [63,64]. 

The results obtained determined that these types of suspensions could be successfully employed in the development of novel antimicrobial agents.

## 3. Materials and Methods

### 3.1. Materials

In order to synthesize the suspensions based on Ciprofloxacin/ Tetracycline embedded in silver doped hydroxyapatite we utilized the calcium nitrate (Ca(NO_3_)_2_·4H_2_O, ≥99.0% purity), diammonium hydrogen phosphate ((NH_4_)_2_HPO_4_, ≥99.0% purity) and silver nitrate (AgNO_3_, 99.0% purity), as well as ciprofloxacin (C_17_H_18_FN_3_O_3_, 98% purity) tetracycline (C_22_H_24_N_2_O_8_, 98% purity) and ethanol (C_2_H_6_O, 99.8% purity). All the precursors were purchased from Sigma Aldrich (St. Louis, MO, USA).

### 3.2. Ciprofloxacin/Tetracycline Embedded in Silver Doped Hydroxyapatite Suspensions

The suspensions based on ciprofloxacin/tetracycline embedded in silver doped hydroxyapatite were synthesized by the adapted co-precipitation method. The solutions based on calcium nitrate with antibiotics (ciprofloxacin/ tetracycline) and diammonium hydrogen phosphate with silver nitrate previously dissolved in water and ethanol were stirred vigorously for 24 h at room temperature (RT). The ratio of [Ca + Ag]/P was adjusted at 5/3, and the concentration of silver was *x*_Ag_ = 0.2 [65,66]. To the phosphate-containing solution, we added drop by drop in the calcium-containing solution and stirred vigorously for 72 h at room temperature. The resulting solution was centrifuged for 15 min at 5000 rpm. The precipitate resulting after centrifugation was washed 5 times by redispersing in water. The precipitate obtained after the last centrifugation was redispersed in water and maturation was performed at RT for another 72 h. The resulting suspensions based on ciprofloxacin embedded in silver doped hydroxyapatite (AgHA-C) and based on tetracycline embedded in silver doped hydroxyapatite (AgHA-T) were analyzed by different techniques and the antimicrobial properties were also investigated.

### 3.3. Characterization Methods

#### 3.3.1. Physico-Chemical Characterization

The mean hydrodynamic diameter of ciprofloxacin/tetracycline embedded in silver doped hydroxyapatite suspensions was determined by dynamic light scattering (DLS) using a SZ-100 Nanoparticle Analyzer (Horiba-SAS France, Longjumeau, France). All measurements were realized three times at 25 ± 1 °C. The stability of ciprofloxacin/ tetracycline embedded in silver doped hydroxyapatite suspensions after maturation was investigated by ultrasonic studies in accordance with previous studies [67,68]. The morphology of the AgHA-C and AgHA-T particles in suspensions was conducted using a scanning electron microscope (FEI Quanta Inspect F, (FEI Company, Hillsboro, Oregon, United States) equipped with an energy-dispersive X-ray attachment (EDX). Using the Energy dispersive X-ray analysis, the local qualitative elemental composition of the samples was identified. The FTIR spectra (Fourier transform infrared spectroscopy) were acquired in ATR (Attenuated total reflectance) using a Universal Diamond/KRS-5 (Waltham, MA, USA) in the range of 400–2000 cm^−1^.

#### 3.3.2. In Vitro Antimicrobial Assay

The antimicrobial activity of the AgHA, AgHA-C, and AgHA-T suspensions and tetracycline and ciprofloxacin were investigated in vitro using the reference *Staphylococcus aureus* ATCC 25923 (ATCC, Old Town Manassas, VA, USA), *Escherichia coli* ATCC 25922 (ATCC, Old Town Manassas, VA, USA), and *Candida albicans* ATCC 10231 (ATCC, Old Town Manassas, VA, USA) microbial strains. The antimicrobial assays were done according to a methodology previously reported in [69,70,71,72], with 0.5 McFarland standard microbial cultures. Afterward, the samples were inoculated using 1.5 mL microbial suspension of a density of 5 × 10^6^ CFU/mL (colony forming units/mL), prepared in phosphate-buffered saline (PBS), and incubated for 24, 48, and 72 h, respectively. As a positive control (C+), free microbial culture was assessed at the same time intervals. Afterward, the suspension was collected at different time intervals (24, 48, and 72 h) and incubated on a LB agar medium for 24 h at 37 °C. The number of CFU/mL was determined for each of the incubated samples with the microbial suspensions. The values of the CFU/mL were determined. The experiments were performed three times and the data were presented as mean ± SD. The statistical analysis was performed using the ANOVA single-factor test.

## 4. Conclusions

The present study aimed to obtain and characterize stable suspensions of AgHA-C and AgHA-T obtained by adapting a coprecipitation method. The evaluation of the antimicrobial properties of AgHA-C and AgHA-T represented another goal of this study. The obtained results showed that the obtained suspensions have very good stability compared to double-distilled water (as reference fluid) and a narrow size distribution. In the FTIR spectra of AgHA-C and AgHA-T, the presence of the two antibiotics led to a slight shift of the vibrational bands specific to pure HA and the appearance of new vibrational bands specific to Ciprofloxacin and Tetracycline. The antimicrobial properties of the AgHA, AgHA-C, AgHA-T as well as Ciprofloxacin and Tetracycline were evaluated against *S. aureus*, *E. coli*, and *C. albicans* microbial strains. The results of the antimicrobial assays depicted that the AgHA-C and AgHA-T exhibited strong inhibitory and microbicide effects on all the tested bacterial strains and for all investigated time intervals. The data also revealed that the antimicrobial properties depended on one hand on the incubation time and on the other hand on the type of microbial strain and antibiotic. The methodology developed in this study represents a safe, fast, and simple way to obtain stable suspensions with antimicrobial properties that could be successfully used in different medical fields.

## Figures and Tables

**Table 1:** The elemental composition estimated from EDS analysis.

Sample	Atomic Composition (%)						
	Ca	P	Ag	O	F	N	C
AgHAp-C	23.87	14.41	0.2	42.35	0.9	1.77	16.5
AgHAp-T	24.39	14.73	0.2	44.29	-	0.98	17.38

**Table 2:** Combined and individual efficacy of tetracycline and ciprofloxacin antibiotics, AgNPs and AgHA against selected microbial strain.

Material	Microbial Strain	Reference
Silver ions	*S. aureus*, *E. coli*, *C. albicans*	[15,17,19,20,35]
Ciprofloxacin	*E coli*, *S. aureus*, *P. aureginosa*	[28,62]
Tetracycline	*C. albicans*, *E coli*	[56,57,58]
AgHA	*C. krusei* 963, *E. coli* ATCC 25922, *K. pneumoniae 2968*, *C. albicans* ATCC 10231	[19,20,35]
AgNPs with ciprofloxacin	*S. aureus*, *A. baumannii*, *and S. marcescens*	[40]
AgNPs with ciprofloxacin, imipenem, gentamycin and vancomycin	*E coli*, *S. aureus*, *M. luteus*, *P. aeruginosa*, *E. faecali*, *A. baumani*, *K. pneumoniae*, *Bacillus* spp.	[63]
AgHA with ciprofloxacin and tetracycline, gentamycin and vancomycin	*E. coli* ATCC 25922 and *S. aureus* 0364	[27,35,59]

## Data Availability

Data is available on demand.
